# Cancer-triggered systemic disease and therapeutic targets

**DOI:** 10.1007/s44178-024-00077-w

**Published:** 2024-03-11

**Authors:** Yihai Cao

**Affiliations:** Department of Microbiology, Tumor and Cell Biology, Karolinska Institute, Solnavägen 9, Stockholm, 171 65 Sweden

**Keywords:** Cancer cachexia, Paraneoplastic syndrome, Metabolic disease, Endocrine disorder, Cancer therapy

## Abstract

Cancer provokes systemic diseases through three possible mechanisms: 1) Distal metastasis in multiple tissues and organs, which directly causes functional damage and impairment of involved organs; 2) Paraneoplastic syndrome (PNS) that affects multiple organ systems, including the endocrine, gastrointestinal, hematologic, neurologic, dermatologic, and ophthalmologic systems; and 3) Cancer cachexia (CCA) or self-wasting syndrome characterized by anorexia, progressive bodyweight loss, adipose atrophy, and muscle atrophy. While cancer metastasis has received considerable attention for comprehensive research, PNS and CCA remain relatively overlooked. At the time of this writing, effective treatments of PNS and CCA in human cancer patients are lacking. This review focuses on discussing mechanistic insights into PNA and CCA and current advances in development of new possible therapeutic interventions.

## Introduction

Approximately 50% of patients with all types of cancer manifest paraneoplastic syndrome (PNS) and/or cancer cachexia (CCA), which are responsible for at least 25% of cancer-related death [1–4]. This life-threatening and devastating cancer-associated systemic disease (CASD) is particularly commonly seen in patients with gastrointestinal, lung, breast, gynecological, hematological, and kidney cancers and affects a vast majority of cancer patients with advanced disease [3, 5]. Although metastatic disease frequently occurs at the advanced stage of malignancy, PNS and CCA are not directly associated with metastases. PNS affects multiple organs and tissues, including neurologic, endocrine, hematologic and dermatologic systems. In some cases, PNS occurs even before cancer diagnosis [5–7].

CCA is featured by progressive bodyweight loss, muscle atrophy, adipose atrophy, and systemic inflammation, which are often associated with anorexia and elevated energy expenditure [8]. CCA alone is responsible for approximate 22% of cancer-related death. Diagnostic criteria of CCA in the clinic are defined by ≥ 5% bodyweight loss within 12 months although some other criteria have been proposed and tailored [9]. Notably, conventional cancer therapies, including chemotherapy and radiation therapy significantly contribute to CCA development in cancer patients [10]. Thus, intensive treatments of CCA patients with these conventional therapeutics may accelerate CCA progression, which affects the quality of life (QOL), survival outcomes, and therapeutic efficacy.

Systemic inflammation has received tremendous attentions as a potential mediator for causing CCA and PNS and inflammation is believed to be the key driving force for altering global metabolism in cancer patients [10, 11]. Cancer cells together with other stromal cells in the tumor microenvironment (TME) release a myriad of inflammatory cytokines that transcriptionally activate catabolism in the targeted cells, including adipocytes, skeletal myocytes, hepatocytes, and hematopoietic cells [12]. Other tumor-derived non-inflammatory factors such as lipase maturation factor (LMF) and proteolysis-inducing factor (PIF) also participate in CCA development by alteration of lipid metabolism and protein catabolism [2]. Tumor-released cytokines and factors often act as systemic hormones to induce high levels of cytokine production in other cell types located in distal tissues and organs, including adipocytes, hepatocytes, gastrointestinal epithelial cells, skeletal myocytes, and cardiomyocytes [6, 11–14]. Collectively, high levels of circulating and tissue-interstitial cytokines and factors contribute to development and progression of CCA and PNS (Table 1). As CCA and PNS have been extensively reviewed elsewhere, this review focuses on discussing new mechanistic insights into CCA and PNS for the purpose to design future effective therapies.

**Table 1:** Examples of factors and cytokines in cancer cachexia and paraneoplastic syndrome

Factor/cytokine^a^	Receptor^a^	Cellular target	Biological function	Reference^b^
TNF-α	TNFR1, 2	Myeloid and other cells	Inflammation etc	[15]
IL-1α/β	IL1R1	Myeloid and other cells	Inflammation etc	[16]
IL-6	IL6R	Myeloid and other cells	Inflammation etc	[17]
VEGF	VEGFR1,2	Endothelial and other cells	Angiogenesis etc	[18]
TGF-β	TGF-βR1,2	Fibroblast and other cells	Fibrosis etc	[19]
BMP	BMP type 1	Osteocyte and other cells	Osteogenesis etc	[20]
IFN-γ	IFNAR	Immune and other cells	Immune response etc	[21]
IL-8	IL8RA	Myeloid and other cells	Inflammation etc	[22]
IL-10	IL-10R1,2	Myeloid and other cells	Inflammation etc	[23]
Myostatin	ACVR2	Various cell types	Myogenesis etc	[24]
Activin	ACVR1,2	Various cell types	Embryogenesis etc	[25]
Leptin	LEP-R	Neuron and other cells	Food intake etc	[26]
Adiponectin	AdipoR1,2	Hepatocyte and other cells	Lipid metabolism etc	[27]
GDF-15	GFRAL	Neuron and other cells	Neurogenesis etc	[28]
IGF	IGFR	Various cell types	Growth etc	[29]
MCP-1	CCR2	Myeloid and other cells	Inflammation etc	[30]
Ghrelin	ghrelinR	Neuron and other cells	Food intake etc	[31]

^a^Abbreviations: *TNF-α* Tumor necrosis factor-alpha, *TNFR* Tumor necrosis factor receptor, *IL-1a/β* Interleukin-1alpha or beta, *IL1R1* Interleukin-1receptor 1, *IL-6* Interleukin-6, *IL6R* Interleukin 6 receptor, *VEGF* Vascular endothelial growth factor, *VEGFR* Vascular endothelial growth factor receptor, *TGF-β* Transforming growth factor-beta, *TGF-βR* Transforming growth factor-beta receptor, *BMP* Bone morphogenetic protein, *IFN-*γ Interferon-γ, *IFNAR* Interferon-α/β receptor, *IL-8* Interleukin-8, IL8RA Interleukin 8 receptor alpha, *IL-10* Interleukin-10, *IL-10R* Interleukin-10 receptor, *ACVR* activin receptor type-2A, *LEP-R* leptin receptor, *GDF-15* Growth/differentiation factor-15, *GFRAL* Glial cell-derived neurotrophic factor family receptor alpha like, *IGF* Insulin-like growth factor, *IGFR* Insulin-like growth factor receptor, *MCP1* Monocyte chemoattractant protein-1, *CCR2* C–C motif chemokine receptor 2,*AdipoR* Adiponectin receptor, *ghrelinR* ghrelin receptor

^b^Owing to an extremely high numbers of references, this article is only allowed to cite a few articles, in most cases review articles. The author apologizes for not being able to cite many important articles published in this field

### Tumor-derived CCA and PNS factors

**Fig. 1:**
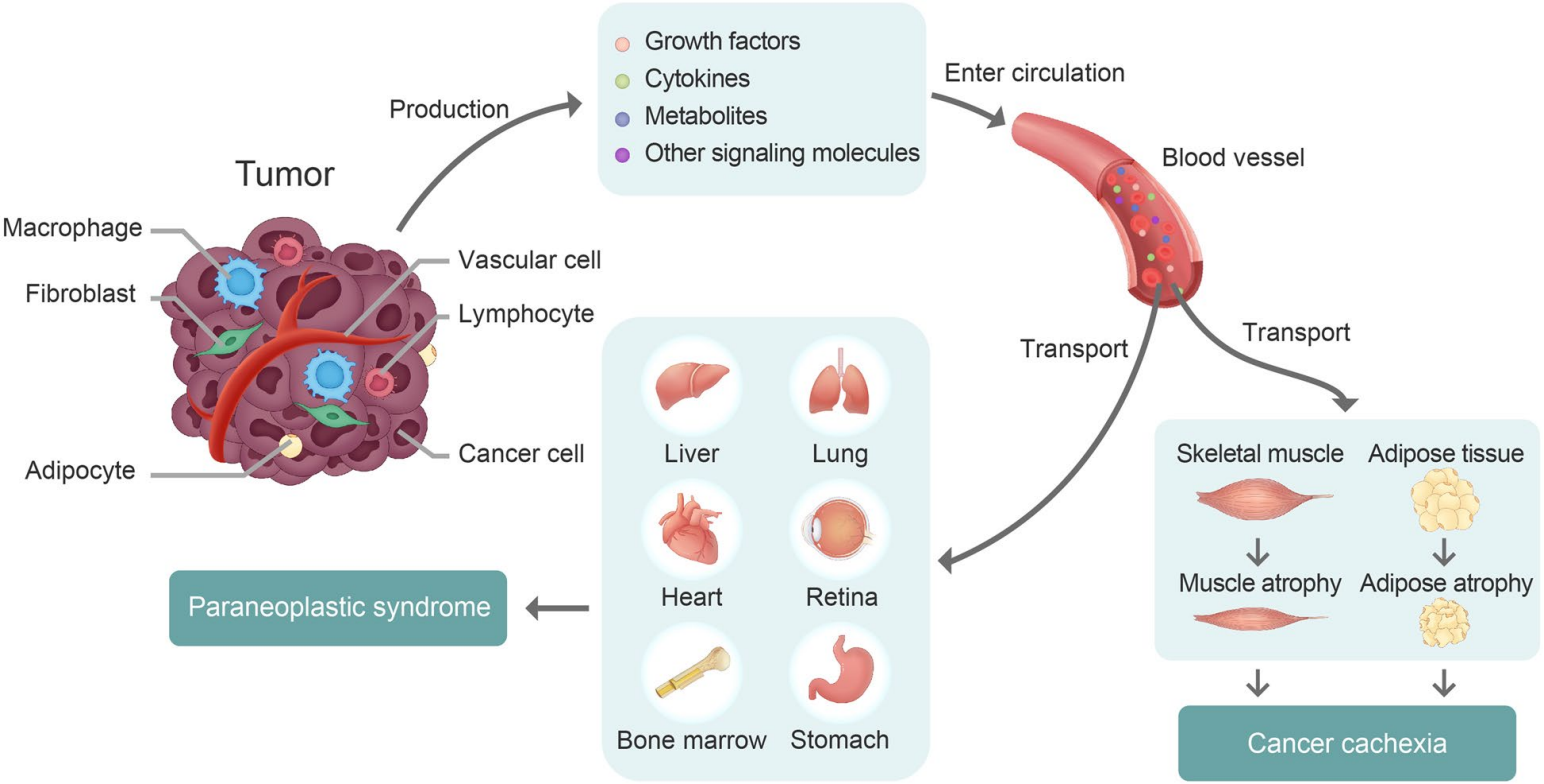
Tumor-derived signaling molecules in systemic cancer disease. Cancer cells and various non-cancerous stromal cells, including fibroblasts, inflammatory cells, vascular cells, immune cells, and adipocytes in the tumor microenvironment (TME) produce high levels of various cytokines and growth factors. In addition to their local functions in TME, these cytokines and growth factors often enter the circulation and act as endocrine-like hormones to target remove tissues and organs, leading to deleterious effects on multiple tissues and organs. The cancer-associated systemic disease (CASD) includes paraneoplastic syndrome (PNS) and cancer cachexia (CCA), manifesting adipose atrophy, muscle atrophy, bodyweight loss, liver dysfunction, and anemia. Targeting the tumor-derived cytokines and growth factors provides an appealing opportunity to developing new drugs for effective treatments of PNS and CCA

In prompting CCA and PNS, a solid tumor acts as a pathological endocrine organ by producing a myriad of cytokines, growth factors, metabolites, and other signaling molecules, which target remote healthy tissues and organs (Table 1) (Fig. 1). Although tumor cells directly synthesize and release these factors and cytokines, other non-cancerous stromal cells in TME, including cancer-associated fibroblasts (CAFs), tumor-associated macrophages (TAMs), vascular endothelial cells (VECs), perivascular cells, lymphocytes, and cancer-associated adipocytes (CAAs) significantly contribute to high production of CCA- and PNS-associated factors [18]. In addition to remotely targeting the CCA- and PNS-affected tissues and organs and reprograming catabolic metabolism and functions, tumor-derived factors act as signaling amplifiers that upregulate expression levels of other cytokines, chemokines and growth factors in various cells [32]. Collectively, tumor- and healthy tissue-derived cytokines and factors induce systemic inflammation and reprogram global metabolism through endocrine and paracrine mechanisms. This review provides in-depth discussion of CCA- and PNS-associated cytokines and growth factors using interleukin-6 (IL-6) vascular endothelial growth factor (VEGF) examples.

### Interleukin-6

IL-6 is a pleotropic cytokine that is frequently expressed at high levels in various tumor tissues and non-malignant tissues in response to tissue damage and infections [33]. IL-6 binds to a IL-6 receptor (IL-6R)-gp130 complex that transduces intracellular signals through the JAK-STAT3 pathway [34]. In addition to the membrane-bound form of IL-6R, IL-6 binds to a proteolytically cleaved soluble IL-6R (sIL-6R). The IL-6-sIL-6R complex binds to the ubiquitously expressed gp130 receptor subunit thought its homodimer to trigger intracellular signaling via the Janus kinase/ signal transducer of activation (JAK/STAT) and mitogen-activated protein kinase (MAPK) pathways. Through this mechanism, IL-6 targets a broad-spectrum of cell types that lack IL-6R expression, a paradigm named the IL-6 trans-signaling [35]. Owing to the IL-6 trans-signaling, IL-6 may serve as the key cytokine to cause sepsis and cytokine storms by systemically targeting multiple tissues and organs.

IL-6 is involved in senescence-associated phenotypes and augmenting skeletal muscle catabolism in the elderly population [36, 37]. Genetic IL-6-overexpressing mouse models show muscle atrophy and similarly IL-6 administration to humans reduces protein synthesis in skeletal muscles [38–40]. However, the IL-6-assocaited muscle atrophy was not observed in experimental rats [41]. Counterintuitively, transient upregulation of IL-6 after physical exercise may mitigate inflammation and increase insulin sensitivity [42]. These findings demonstrate that the role of IL-6 in regulation of muscular catabolism is far more complex, perhaps in the pathophysiological context-dependent manner.

Although IL-6 is synthesized and released by many cell types in various organs, it has been estimated that white adipose tissue (WAT) alone contributes to approximate 35% of total IL-6 molecules in human blood. In obese individuals, circulating IL-6 levels are elevated largely due to significant release from visceral WAT [43, 44]. It seems that both inflammatory macrophages and adipocytes are the main cellular sources for IL-6 production [44]. High levels of IL-6 exist in patients with various types of cancer and is often reversely correlated with poor survival [45]. Adipocyte specific deletion of the Il-6 gene in mice reduced circulating IL-6 protein levels by approximate 40%, demonstrating adipocytes as the key cellular source of IL-6 [44, 46]. Additionally, genetic deletion of IL-6 in mice also mitigates inflammation in obese adipose tissues [44, 46].

IL-6 promotes tumor growth and metastasis through several mechanisms, including inflammation, angiogenesis, epithelial-mesenchymal transition (EMT), and metabolic reprograming [47]. IL-6 induces polarization of M2-macrophages, which in turn promotes cancer metastasis [48, 49]. IL-6-induced tumor neovascularization and vascular remodeling not only facilitate cancer metastasis [47], but also significantly modulate the endocrine function of IL-6 by regulating vascular transport. Circulating IL-6 levels have been correlated with anorexia [50], a common caloric deficiency associated with CCA. One of the mechanisms that underlie the IL-6-triggered excessive energy expenditure, adipose atrophy, and bodyweight loss in cancer hosts is that IL-6 promotes non-shivering thermogenesis (NST) by activation of thermogenic adipose tissues (TATs), including brown adipose tissue (BAT) and browning WAT [44, 51, 52]. TAT activation has been linked to CCA development in preclinical models [53]. IL-6 also induces liver dysfunction, a transitional stage of CCA development, by augmenting ER stress and cellular apoptosis [54, 55].

Therapeutic targeting the IL-6 signaling for treatment of CCA has been evaluated in both preclinical models and clinical trials [56]. In a mouse CCA model, blocking IL-6R by tocilizumab, an anti-IL-6R neutralizing antibody, alleviates CCA [57]. Similarly, clazakizumab, a humanized rabbit monoclonal antibody against IL-6, produces encouraging results in a Phase II clinical trial by preventing muscle wasting and improving hematopoiesis [58]. In another Phase II trial, simultaneous targeting IL-6 and TNF-α by a broad-spectrum agent also improves bodyweight gain in patients with advanced cancer disease [59].

### VEGF

VEGF is one of the key angiogenic factors utilized by various tumors to stimulate neovascularization [60, 61]. Although several other structurally and functionally related members, including VEGF-B, VEGF-C, VEGF-D, and placental growth factor (PlGF) exist within the VEGF family [62], VEGF, also named VEGF-A, as the prototype member displays broad biological functions [11, 63]. Biological functions of VEGF include, but limited to, angiogenesis, vascular permeability, vascular remodeling, endothelial survival, neurotrophic functions, inflammation, endocrine regulation, hematopoiesis, metabolism, and embryogenesis [11]. Most of these VEGF-stimulated functions are mediated by VEGF receptor2 (VEGFR2), a transmembrane tyrosine kinase receptor, although VEGFR1, often acting as a decoyed receptor, also participates in mediating some of these functions [62].

Compared with their adjacent heathy tissues where tumors originate, tumor tissues almost always express higher levels of VEGF [64]. There are several possible mechanisms that underlie high VEGF production in tumors, including: 1) Genetic mutations in cancer cells as drivers for elevating VEGF expression. For example, genetic mutations of von Hippel-Lindau (VHL) in renal cell carcinoma (RCC) lead to exceptionally high expression of VEGF through stabilization of hypoxia-inducible factor (HIF)-1α that transcriptionally controls VEGF mRNA expression [65]. Another example is Kirsten rat sarcoma virus (KRAS) mutations, commonly existing in epithelial carcinomas, which markedly upregulate VEGF expression [66]; 2) Stromal cellular components in TME such as inflammatory cells and fibroblasts significantly contribute to VEGF production [67, 68]; 3) Hypoxia. Despite enrichment of blood supply, most solid tumors experience various degrees of hypoxic insults, often owing to the structural malformation and dysfunctional blood vessels [69]. Hypoxia upregulates VEGF expression via the HIF-1α-triggered transcription activation of the VEGF gene promoter [70, 71]; and 4) Accumulation of metabolites. TME often exhibits acidosis owing to accumulation of acidic metabolites from highly proliferating tumor cells and stromal cells [72]. These acidic metabolites augment VEGF production.

VEGF is synthesized in various cells and exists in various isoforms due to alternative splicing of its mRNA [73]. While high molecular forms of VEGF molecules are often sequestered in the local tissue where they are produced by interacting with heparan sulfate proteoglycans (HSPGs) in the extracellular matrix and on the cell surface, smaller VEGF isoforms can diffuse into the circulation and act as endocrine hormones [74]. In preclinical cancer models, high expression of VEGF in tumor cells induces a PNS-like systemic disease, manifesting multiorgan dysfunctions [6]. Circulating VEGF molecules preferentially target sinusoidal vasculatures in bone marrow (BM), liver and spleen, and fenestrated vasculatures in endocrine organs such as those located in the pancreatic islet, adrenal gland, and thyroid [6]. For example, subcutaneous implantation of an VEGF-overexpressing tumor in mice induces hepatomegaly, splenomegaly, BM-associated anemia, and endocrine dysfunctions by altering vascular architectures and structures [6, 18]. These preclinical findings are highly relevant to human patients with certain types of cancer. As discussed above, RCC patients carrying VHL mutations often have high circulating levels of VEGF and manifest PNS [6, 7, 64, 65]. In an early study, autopsy analysis of cancer patients demonstrated that about 20% of RCC patients had hepatomegaly owing to vascular dilation [75]. Although VEGF was not discovered at that time, this study speculated that RCC tumors produce X factor(s) that ultimately targets remote tissues and organs for causing PNS.

VEGF plays a crucial role in regulation adipose metabolism [76–81]. Activation of thermogenesis in TATs by cold exposure or β3-adrenoceptor agonists is dependent on the VEGF-induced adipose angiogenesis [78–83]. Loss-of-function experiments by a pharmacological anti-VEGF approach inhibits TAT activation and NST metabolism [79, 84]. Likewise, genetic specific deletion of the Vegfr2 gene in endothelial cells completely abrogates the cold exposure-induced adipose angiogenesis and browning of TATs [84]. Interestingly, inhibition of VEGFR1 alone markedly induces a browning phenotype of WAT and thermogenic activation of TATs [77], indicating the negative role of VEGFR1 in regulation of adipose NST metabolism. In contrast, delivery of VEGF to WAT augments a local browning phenotype [84]. Noticeably, VEGFRs are primarily expressed in vascular endothelial cells, but not in adipocytes. These findings indicate that the adipose vasculature plays a pivotal role in controlling adipocyte metabolism. Along the CCA trajectory, tumor-derived circulating VEGF molecules likely to contribute to adipose atrophy and bodyweight loss by stimulating energy expenditure.

Anti-VEGF-based antiangiogenic drugs are broadly used in the clinic for treatment of various cancers in human patients [63]. Although there has been lacking sufficient clinical data to convincingly correlate improvement of PNS and CCA by these drugs with clinical outcomes, I reasonably speculate that improvement of the systemic cancer disease by anti-VEGF drugs likely contributes to prolonged survivals of cancer patients in either monotherapy and combination therapy settings. This view is supported by the fact that survival improvement by anti-VEGF drugs may not be necessarily correlated with reduction of the tumor size [7], a clinical parameter commonly used to determine anticancer effects. A preclinical study shows survival improvement by an anti-VEGF drug is not correlated with decreasing of the tumor mass, but rather through the mechanism of improving systemic cancer disease [6]. This important issue warrants future clinical validation.

### Conclusions and perspectives

Malignancy is not a local disease that only affects the tissue where tumors originate, but rather a systemic disease that in principle affects multiple organs. Although metastasis significantly attributes to the most of cancer-related death, CCA and PNS are responsible for high mortality of cancer patients. This mini-review discusses IL-6 and VEGF as two examples of tumor-derived cytokines and growth factors in causing PNS and CCA. Obviously, multiple cytokines and growth factors are involved causing the systemic cancer disease (Table 1). In addition to their own receptor-mediated signaling pathways, cytokines and growth factors often interact each other to amplify their biological signals or synergistically act on their targeted cells. Thus, disruption of the interaction loop by a specific drug that targets one cytokine/growth factor may produce profound impacts on other signaling pathways. Taken IL-6 and VEGF as examples, inhibition of IL-6 or its receptors may downregulate VEGF production from cancer cells and inflammatory cells because IL-6 is known to instigate VEGF production. Thus, blocking IL-6 by drugs may indirectly affect VEGF-triggered PNS and CCA. Like other therapeutic regimens, effective treatments of PNS and CCA may embroil complex approaches by combining drugs with different principles and molecular targets of cancer cells and other host cells (Table 2). Unlike drugs targeting cancer cells, future drugs for treating PNS and CCA may aim to normalize functions of the cancer-affected tissues and organs (Table 2). Thus, systemic rather than local treatments should be considered for drug development. As patients with systemic cancer disease often occur at the advanced stage of malignancy, they are often intolerable to toxic drugs with severe adverse effects. In particular, if combinations of drugs are considered for clinical use, the combined drug toxicities should be minimized and tolerable for cancer patients with advanced cancer disease. Despite the well-accepted reduction of the tumor mass as a surrogate marker for assessing anticancer effects, survival improvement is the ultimate criterion for determining clinical outcome and for receiving the FDA approval of anticancer drugs. Again, clinical benefits of anti-PNS and anti-CCA drugs should be especially determined by survival improvement (Table 2). Although improvement of QOL by drugs should also be taken into consideration, the criteria for QOL improvement by drugs are far more byzantine, which vary considerably between individual cancer patients. Together, effective drugs for treatment of PNS and CCA are not clinically available at the time of this writing and they are urgently needed for improving survival and QOL of cancer patients.

**Table 2:** Clinical development of anti-cancer cachexia drugs

Drug	Target	Phase	Human subject	Clinical outcome	Trial number
Etanercept	TNF-α^a^	II	Cancer patients	Safety, bodyweight	NCT00201838
Infliximab	TNF-α	Pilot/II	Cancer patients	Safety, bodyweight	[85]
Pentoxifylline	TNF-α	Pilot	Cancer patients	Safety, bodyweight	[86]
Clazakizumab	IL-6	II	Healthy volunteers	Safety, bodyweight	NCT04348500
Bermekimab	IL-1α	I	Cancer patients	Safety, bodyweight	NCT03207724
LY2495655	Myostatin	II	Cancer patients	Safety, bodyweight	NCT01505530
BYM338	ACVR2	II	Cancer patients	Safety, bodyweight	NCT01669174
Enobosarm	SARM	III	Cancer patients	Bodyweight loss	NCT01355484,97
Formoterol	LABA	I/II	Cancer patients	Safety, bodyweight	[87]
Espindolol	β-adrenoceptor	II	Cancer patients	Safety, bodyweight	NCT0123807
Thalidomide	TNF−⍺	Pilot	Cancer Patients	Bodyweight loss	[88]
Olanzapine	Dopamine	III	Cancer patients	Appetite	NCT4939090
Ruloxitinib	JAK/STAT	Pilot	Cancer patients	Safety, anorexia	NCT04906746
Ponsgromab	GDF-15	II	Cancer patients	Safety, PK	NCT05546476
Ponsgromab	GDF-15	Ib	Cancer patients	Safety, PK	NCT04299048
NGM120	GDF-15	I/II	Cancer patients	Safety, PK	NCT04068896
AV380	GDF-15	I	Healthy volunteers	Safety, PK	NCT04815551
CTL002	GDF-15	I/II	Cancer patients	Safety, PK	NCT04725474
PF-07258669	MC4R	I	Healthy volunteers	Safety, PK	NCT04628793
TCMCB07	MC4R	I	Healthy volunteers	Safety, PK	NCT05529849
Anamorelin	ghrelinR	III	Cancer patients	Bodyweight, anorexia	NCT03743064
Anamorelin	ghrelinR	III	Cancer patients	Bodyweight, anorexia	NCT03743051
Anamorelin	ghrelinR	II	Cancer patients	Bodyweight, anorexia	NCT04844970

^a^*Abbreviations: TNF-*⍺ Tumor necrosis factor-alpha, *IL-6* = Interleukin-6, *IL-1β* Interleukin-1beta, *ACVR* Activin receptor type-2A, *SARM* Selective androgen receptor modulator, *LABA* Long-acting β2 agonist, *JAK/STAT* Janus kinase/signal transducer of activation,GDF-15 Growth/differentiation factor-15, *MC4R* Melanocortin 4 receptor, *ghrelinR* Ghrelin receptor

## Data Availability

Not applicable.

## Abbreviations

ACVR: Activin receptor type-2A
AdipoR: Adiponectin receptor
BAT: Brown adipose tissue
BM: Bone marrow
BMP: Bone morphogenetic protein
CAA: Cancer-associated adipocyte
CAF: Cancer-associated fibroblast
CASD: Cancer-associated systemic disease
CCA: Cancer cachexia
CCR2: C-C motif chemokine receptor 2
EMT: Epithelial-mesenchymal transition
GDF-15: Growth/differentiation factor-15
GFRAL: Glial cell-derived neurotrophic factor family receptor alpha like
ghrelinR: Ghrelin receptor
HIF: Hypoxia-inducible factor
HSPG: Heparan sulfate proteoglycan
IFN-γ: Interferon-γ
IFNAR: Interferon-α/β receptor
IGF: Insulin-like growth factor
IGFR: Insulin-like growth factor receptor
IL-1: Interleukin-1
IL1R1: Interleukin-1receptor 1
IL-6: Interleukin-6
IL-6R: IL-6 receptor
IL-8: Interleukin-8
IL8RA: Interleukin 8 receptor alpha
IL-10: Interleukin-10
IL-10R: Interleukin-10 receptor
JAK: Janus kinase
KRAS: Kirsten rat sarcoma virus
LABA: Long-acting β2 agonist
LEP-R: Leptin receptor
LMF: Lipase maturation factor
MAPK: Mitogen-activated protein kinase
MC4R: Melanocortin 4 receptor
MCP1: Monocyte chemoattractant protein-1
mRNA: Messenger ribonucleotide acid
NST: Non-shivering thermogenesis
PIF: Proteolysis-inducing factor
PlGF: Placental growth factor
PNS: Paraneoplastic syndrome
QOL: Quality of life
RCC: Renal cell carcinoma
SARM: Selective androgen receptor modulator
sIL-6R: Soluble IL-6R
STAT: Signal transducer of activation
TAT: Thermogenic adipose tissue
TAM: Tumor-associated macrophage
TGF-β: Transforming growth factor-beta
TGF-βR: Transforming growth factor-beta receptor
TME: Tumor microenvironment
TNF-α: Tumor necrosis factor-alpha
TNFR: Tumor necrosis factor receptor
VEC: Vascular endothelial cell
VEGF: Vascular endothelial growth factor
VEGFR: VEGF receptor
VHL: Von Hippel-Lindau
WAT: White adipose tissue
